# A Rare Case of Abdominal Wall Schwannoma

**DOI:** 10.7759/cureus.111498

**Published:** 2026-06-25

**Authors:** Mary E Londoño, Vanessa Le, Andrea J Lee, Oliver Eng, Michael P O'Leary

**Affiliations:** 1 General Surgery, University of California Irvine Medical Center, Orange, USA; 2 Surgical Oncology, University of California Irvine School of Medicine, Irvine, USA; 3 Pathology, Tibor Rubin VA Medical Center, Long Beach, USA; 4 Surgical Oncology, University of California Irvine Medical Center, Orange, USA

**Keywords:** abdominal wall schwannoma, benign neoplasm, cellular schwannoma, histopathology, merlin, peripheral nerve sheath tumor, rare case report, s100-positive tumor, soft tissue tumor, surgical excision

## Abstract

Schwannomas are relatively uncommon benign tumors of the peripheral nerve sheaths most commonly found in the head, neck, and extremities. Very few reported cases of abdominal wall schwannomas exist.

We report the case of a 71-year-old man with a left-sided abdominal wall mass who subsequently underwent surgical excision with final histopathology demonstrating a cellular schwannoma. Histopathological analysis confirmed the diagnosis, revealing immunoreactivity for S100, SOX10, and vimentin while being negative for smooth muscle actin, CD34, and desmin. The Ki-67 proliferative index was 5%. These findings are consistent with a schwannoma.

In this study, we emphasize the importance of considering schwannoma when evaluating soft tissue masses, even in atypical locations. This unusual finding adds to the available limited literature describing abdominal wall schwannomas.

## Introduction

Schwannomas, also called neurilemmomas, are rare but benign tumors of the peripheral nerve sheath that consist exclusively of Schwann cells [1,2]. Despite their rarity, schwannomas remain the most common benign nerve sheath tumor, with approximately 5% incidence in adults [1-4]. Notably, abdominal wall schwannomas are an even rarer subtype, with only 10 case reports to date available in the English language literature [1,2,5,6]. Because abdominal wall schwannomas are so infrequently described, this case serves to broaden the differential diagnostics, recognition, and understanding of this unique pathology.

Schwannomas usually occur spontaneously, but can occur in relation to tumor syndromes, namely, neurofibromatosis type 2 (NF2), schwannomatosis, or Carney's complex [3,7]. Unlike neurofibromas found with NF2, schwannomas do not contain a nerve entering and exiting the mass but are eccentric to a nerve [3]. On gross examination, they appear as well-circumscribed, encapsulated masses. On histology, they have a characteristic morphology of tightly organized spindle cells (Antoni A) that abruptly change to loose, lower-density cells (Antoni B) with immunohistochemistry stains positive for S100 and SOX10 [3,7]. Only one subtype of schwannoma, the "ancient” schwannoma, displays degenerative changes [8].

Similar to other nerve sheath tumors, schwannomas develop in areas with high densities of peripheral nerve endings, most commonly in the retroperitoneum, mediastinum, head and neck, and extremities [2,3,6,9]. They are often discovered incidentally, as they are typically asymptomatic; however, symptoms such as paresthesia, swelling, and pain can occur due to compression of local anatomy [1,2,10].

Diagnosis typically requires surgical excision to distinguish from other benign soft tissue masses, but it also serves as the standard treatment for schwannomas [3]. Given the low rate of malignancy and recurrence, enucleation and partial excision have also been considered appropriate treatment options if excision is difficult and the patient's symptoms are improved with partial excision [1,3,5].

We report here the case of a 71-year-old man with a symptomatic, abdominal wall cellular schwannoma. Given the paucity of publications on abdominal wall schwannomas, we hope to elaborate on the existing understanding of this unusual pathology and propose potential areas of future research.

## Case presentation

We report the case of a 71-year-old man with a history of prior cerebrovascular accident, hyperlipidemia, and hypertension who presented to the surgical clinic with complaints of a palpable left-sided abdominal wall mass that he had first noticed approximately two months prior. He complained of mild pain in and around the mass with direct pressure for a prolonged period. He characterized the pain as a burning or "tingling" pain. He had no history of prior abdominal surgeries. On examination, the mass was noted to be firm, well-circumscribed, and freely mobile, roughly 2 cm in size without overlying skin changes.

**Figure 1:**
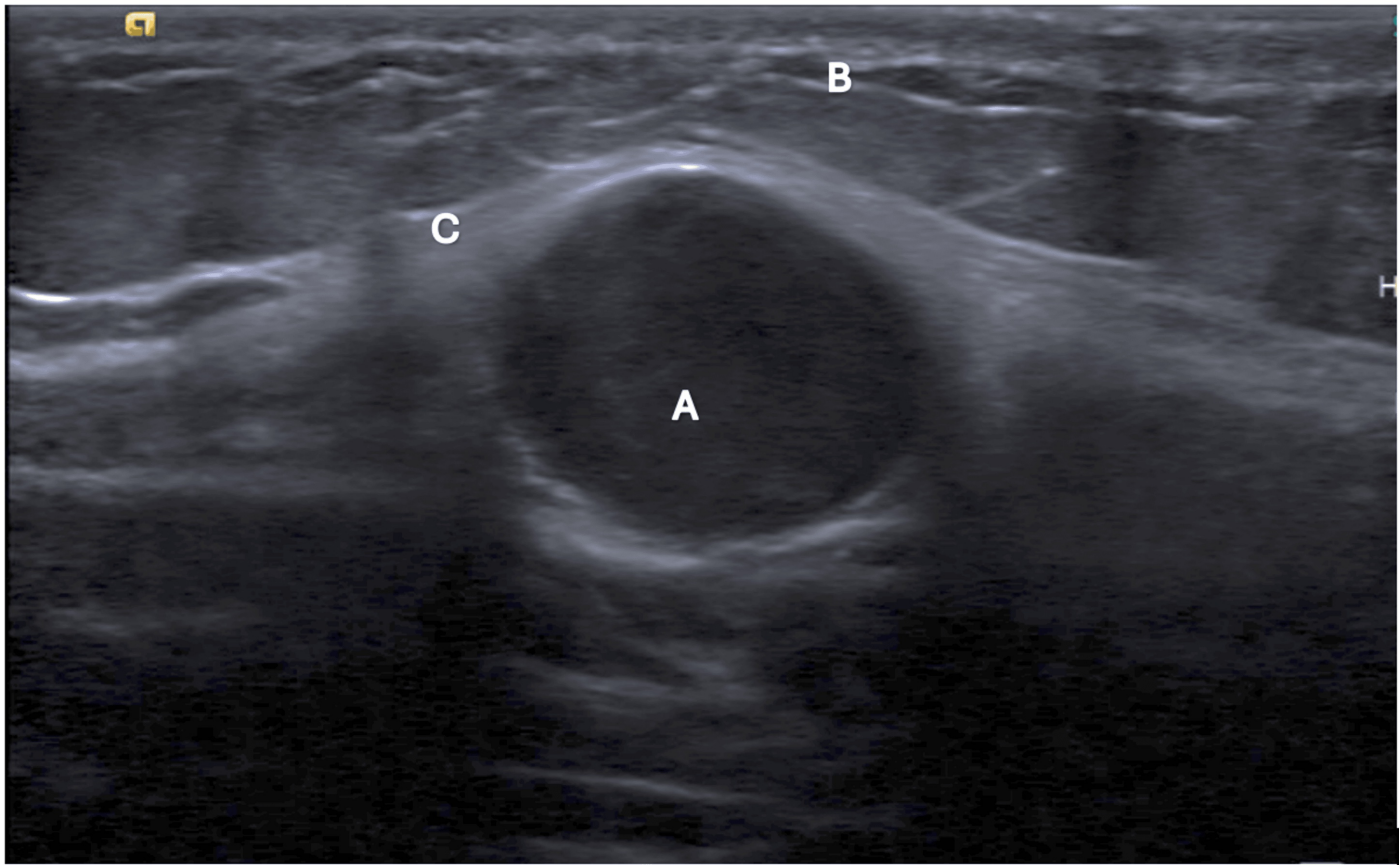
A preoperative abdominal soft tissue ultrasound of the mass Ultrasound demonstrates a nonspecific, hypoechoic 1.9 cm structure (A) in the subcutaneous tissue (B), positioned slightly deeper than expected for a typical epidermal inclusion cyst, i.e., deep to the fascia (C). Malignancy could not be excluded based on the ultrasound.

He underwent an appropriate workup, including a soft tissue ultrasound of the abdominal wall that demonstrated a nonspecific 1.9 cm structure in the left anterolateral subcutaneous tissue, positioned slightly deeper than expected for a typical subcutaneous mass or cyst (Figure 1). Given the patient's symptoms, malignant differential, and imaging characteristics, surgical excision was planned.

**Figure 2:**
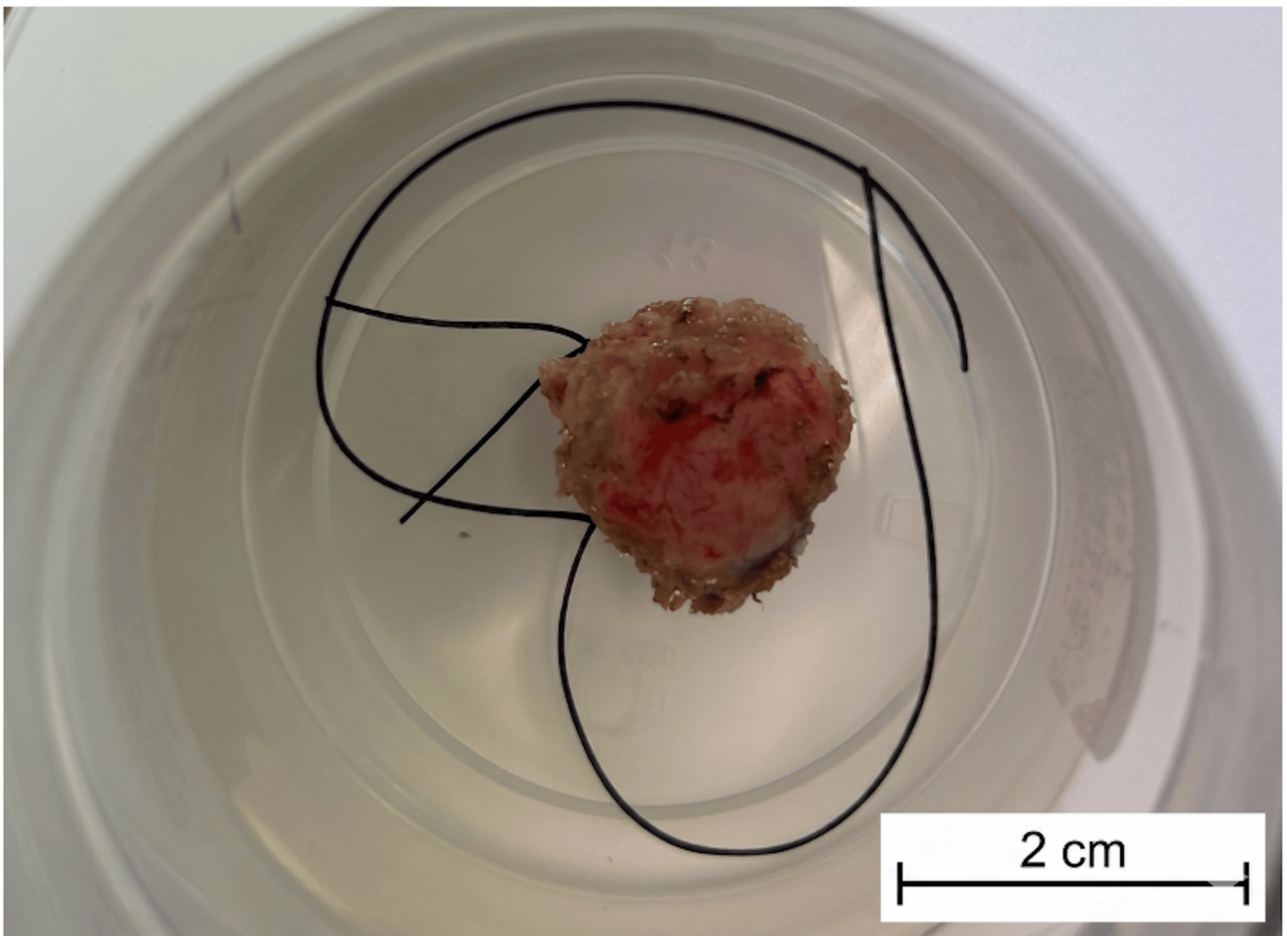
Intraoperative imaging of the gross surgical specimen Sutures mark orientation (short, superior; long, left lateral). The specimen was firm and rubbery and appeared encapsulated (capsule kept intact) which was inconsistent with lipomatous lesions. It measured 2 cm approximately; measurement to scale in bottom right for reference.

The patient underwent an elective excision of the left-sided abdominal mass. Intraoperatively, the mass was found to be intramuscular, within the external abdominal oblique. Gross inspection of the mass in the operating room revealed a well-circumscribed nodular mass that was firm to palpation, firmer than a lipomatous mass (Figure 2). Based on the involvement of muscle and the appearance of the specimen, the differential included benign etiologies; however, malignant liposarcoma or leiomyosarcoma was also suspected. The specimen's orientation was marked and submitted for pathological analysis. The patient recovered well postoperatively without complications and was discharged the same day.

**Figure 3:**
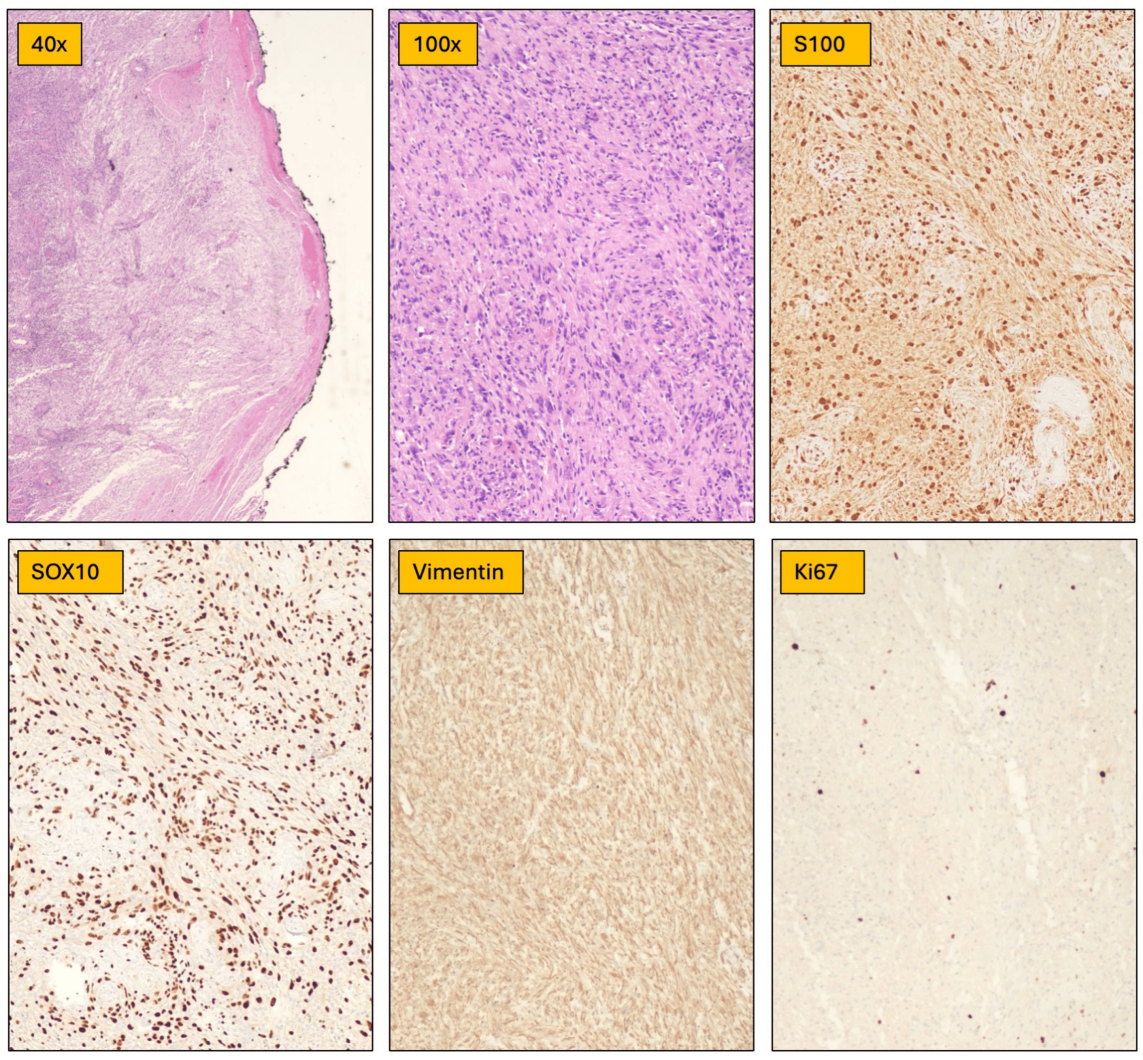
H&E staining of the specimen H&E sections show an encapsulated neoplasm composed of cytologically bland spindle cells arranged in short fascicles. The tumor cells are immunoreactive for S100, SOX10, and vimentin. The Ki-67 proliferative rate is approximately 5%. H&E: hematoxylin and eosin

Histopathological analysis demonstrated a cellular schwannoma with focal degenerative change. Immunohistochemical staining confirmed the diagnosis, revealing immunoreactivity for S100, SOX10, and vimentin while being negative for smooth muscle actin, CD34, and desmin (Figure 3). The Ki-67 proliferative index was 5%. These findings were consistent with a cellular schwannoma. The patient was seen in follow-up without concern or recurrence of symptoms.

## Discussion

Schwannomas are rare but benign tumors of the peripheral nerve sheath that consist exclusively of Schwann cells [1,2]. While they are relatively rare tumors, they are even more rarely found in the abdominal wall [2,5]. They tend to appear in areas of the body with high density of nerve endings, such as the face, cranial nerves, and extremities, which likely contributes to the rare presentation on the trunk [2,3,6,9]. On the abdomen, they are often asymptomatic with incidental discovery, but may present with symptoms due to compression on adjacent nerves [1,2,10]. Among the 10 reported abdominal wall schwannomas, only six presented with symptoms of pain similar to our patient [6]. In terms of diagnostics, in all cases, the lesions appeared hypoechoic on ultrasound, a subtle distinction when compared to other soft tissue masses [1,4,6]. Because they are typically asymptomatic, it is a possibility that the low published rates of abdominal wall schwannomas are related to underdiagnosing as opposed to actual low incidence and prevalence. This suggests that greater attention to imaging and exam details is indicated for accurate diagnosis.

The histological findings of our patient's mass were consistent with a schwannoma with some focal degenerative change. The only subtype of schwannoma that typically demonstrates degenerative changes is the "ancient" schwannoma [8]. In order to be classified as an "ancient" schwannoma, the tumor must exhibit calcification, interstitial fibrosis, and cystic or vascular hyalinization, caused by long-standing presence [7,10]. Despite our patient's age, he had only had symptoms for several months, suggesting a relatively new mass. This tumor had very minimal focal degenerative changes, precluding it from the category of "ancient"; however, if left untreated, it would likely have progressed to an ancient cellular schwannoma. In the established literature, approximately 40-50% of abdominal wall schwannomas have been ancient schwannomas [1,6].

Literature review of all case reports demonstrates that most, if not all, documented abdominal wall schwannomas were found in either the musculature of the right iliac fossa or, as in our patient, the left anterolateral upper quadrant [6]. Both locations involve abdominal musculature which develops from the hypaxial myotome. This commonality may be suggestive of a potential embryologic link between peripheral nerve pathways and the abdominal wall musculature. Defects in merlin (also known as schwannomin) have been associated with the development of schwannomas in both sporadic and genetically associated schwannoma formation [7]. Likewise, merlin has been found to be a key regulator of embryological muscle development with varying expression in migratory mesoderm and axial mesoderm [7,11,12]. More research is needed to determine if merlin's role in hypaxial myotome development (ventral abdominal wall musculature) contributes to the sites of abdominal wall schwannoma formation [7,12]. That said, if a soft tissue mass is identified in the abdominal wall musculature, schwannoma should be placed on the differential alongside more common masses such as lipomas, given the location.

The gold standard of treatment for abdominal wall schwannoma is surgical excision if symptomatic or small [3,5]. It is only via histological evaluation that a sure diagnosis of schwannoma can be made. The low rate of recurrence and malignancy makes surgical excision an optimal diagnostic and curative management [3,5,7].

## Conclusions

This case report highlights a rare presentation of an abdominal wall schwannoma in a 71-year-old man, contributing to the limited literature on these abnormal occurrences of schwannomas. Through clinical and histopathologic evaluation, this case underscores the importance of considering schwannomas in the differential diagnosis of soft tissue masses, even when in atypical locations. Our discussion of schwannoma pathology and potential embryology provides a foundation for further inquiry into the possible etiology of these atypical presentations. We propose that future research should focus on the mechanisms underlying abdominal wall schwannoma formation to improve diagnostic and therapeutic approaches.
